# Trazodone and patient outcomes in dementia—Limitations of naturalistic cohort data

**DOI:** 10.1002/gps.5777

**Published:** 2022-08-03

**Authors:** Emad Sidhom, Mc Stephen Padilla, Jonathan Lewis, Simon White, John T. O’Brien, Giovanna R. Mallucci, Benjamin R. Underwood

**Affiliations:** ^1^ UK Dementia Research Institute and Department of Clinical Neurosciences University of Cambridge Island Research Building Cambridge Biomedical Campus Cambridge UK; ^2^ Cambridgeshire and Peterborough NHS Foundation Trust Windsor Unit Fulbourn Hospital Cambridge UK; ^3^ University of Cambridge School of Clinical Medicine Cambridge Biomedical Campus Cambridge UK; ^4^ Cambridge and Peterborough NHS Foundation Trust Cambridge UK; ^5^ Department of Psychiatry University of Cambridge Cambridge UK

**Keywords:** Alzheimer's disease, dementia, naturalistic cohort, trazodone, unfolded protein response

## Abstract

The unfolded protein response has been increasingly implicated as an important pathological pathway and target for therapeutic intervention in neurodegeneration. The licensed antidepressant trazodone is one drug which has been proposed to act on this pathway and may therefore be a potential therapy. Previous examination of existing data for patients with dementia prescribed trazodone did not find a signal suggesting a disease modifying effect. Here we add to that literature by examining the electronic patient record of patients with dementia in Cambridgeshire UK. We found that trazodone is rarely prescribed and where it is used it is at a dose less than half that predicted to be disease modifying. We also found that patients prescribed trazodone had higher levels of neuropsychiatric symptoms and were relatively late in the disease course, likely beyond the optimal point for therapeutic intervention. We suggest it is therefore premature to discard potential therapies based on observational data alone, particularly when experimental medicine approaches to examine the effects of trazodone are feasible.

## Key points

- Trazodone is rarely prescribed for patients with dementia
- Doses of trazodone used for patients with dementia are generally low, an average of 75mg in this study
- Patients with dementia who are prescribed trazodone are different to those who are not, including higher rates of neuropsychiatric symptoms which are a common reason for prescribing
- These findings suggest naturalistic observational data cannot be relied on to confirm or refute any disease modifying effect of trazodone.

1

The unfolded protein response (UPR) is a key pathological pathway in neurodegeneration, both in animal models and in human post‐mortem brains of patients with Alzheimer's disease.^1^ The UPR has therefore emerged as a therapeutic target for dementia.^2^ One licensed drug that targets the UPR is the antidepressant trazodone, which restores memory and prevents neuronal loss in mouse models of these diseases.^3^ Trazodone is therefore a potential disease‐modifying therapeutic agent for dementia. This has led to interest in ascertaining evidence for any disease‐modifying effects of trazodone in patients with dementia from naturalistic cohorts^3^, ^4^

Sommerlad and colleagues examined the cognitive outcome in patients with dementia prescribed trazodone (406 patients; mean duration 2.2 years) using routinely‐collected patient data in the UK.^5^ Trazodone treated individuals had mildly worse cognitive outcome, measured by Mini Mental State Examination (MMSE) by 0.26 points, than patients with dementia treated with citalopram. The authors conclude that trazodone is not disease‐modifying in dementia. This is a fair interpretation of the data, in the context of its current routine clinical use. However, it is worth examining that clinical use in more detail before definitive conclusions can be drawn with respect to all patients with dementia.

We used the Cambridgeshire and Peterborough NHS Trust (CPFT) research database for Cambridgeshire, UK, to search the anonymized electronic patient record between 01/2013 and 06/2021. The clinical service covers a population of c0.86 million people; including 165,000 people over 65. 11,637 patients with a diagnosis of dementia were identified. We used a natural language processing tool to identify those with ‘trazodone’ in their record which returned 254 results. Each was individually reviewed to identify patients who had actually been prescribed trazodone. This resulted in 157 patient records in which individuals were exposed to trazodone, compared to a control group of 1570 patients with a diagnosis of dementia, where trazodone was not mentioned in the records. Groups were compared using Wilcoxon test for continuous and Fisher's exact test for categorical variables. The results are shown in Table 1.

**TABLE 1:** Patients with dementia on trazodone compared to patients not taking trazodone

Item	Trazodone	Control group	*p*‐value
Total number of patients	157	1570	
Gender	86(M)/71(F)	635(M)/935(F)	<0.001
Mean age	81.3	84.8	<0.001
Total alive/deaths	69/88	717/853	0.762
Mean age (living)	78.83	83.44	<0.001
Mean age at death	83.10	85.82	<0.001
Mean years from diagnosis to analysis for all patients	3.83	3.01	0.018
Mean years from diagnosis to death	2.49	2.25	0.108
Mean years from diagnosis to analysis for patients still alive	4.55	3.93	0.008
Mean trazodone dose (mg)	75	N/A	
Cognitive assessments at time of prescription			
Mean MMSE	22.25 (60 patients)	20.8 (552 patients)	0.235
Mean ACE	67 (80 patients)	66.7 (797 patients)	0.854
Mean Mini‐ACE	12.28 (21 patients)	13.3 (246 patients)	0.469
Mean MoCA	12 (1 patients)	14 (15 patients)	NA
Average initial HoNOS			
Behaviour	1.32	0.58	<0.001
Self‐harm	0.05	0.05	0.688
Substance abuse	0.10	0.06	0.413
Cognitive	2.47	2.22	0.034
Disability	1.54	1.54	0.978
Hallucinations	0.44	0.42	0.604
Depressed	0.76	0.67	0.280
Relationships	0.89	0.52	<0.001
Other	1.54	0.89	<0.001
ADL	2.06	1.68	0.011
Living conditions	0.42	0.26	0.087
Occupation	1.13	0.66	<0.001
Total	12.76	9.60	<0.001

Abbreviations: ACE, Addenbrooke's cognitive examination; HoNOS, nation outcome scale scores; MMSE, mini mental state examination; MoCA, Montreal cognitive assessment.

Trazodone was uncommonly used: in just 1.3% (157/11,637) of patients with a dementia diagnosis. The mean dose prescribed was 75 mg (25–300 mg). Where trazodone is prescribed, it is used for neuropsychiatric features: behavioural disturbance (17/156), insomnia (10/156), depression (<10/156) and anxiety (<10/156) in patients with moderate disease (mean MMSE 22.25; SD = 5.051), Addenbrooke's Cognitive Examination (ACE) 67 (SD = 33.779, mini‐ACE 12.28 (SD = 5.892), Montreal Cognitive Assessment (MoCA) of 12) and a mean duration of illness significantly longer than controls (3.83 vs. 3.01 years). Patients treated with trazodone had significantly higher mean Health of the Nation Outcome Scale scores (HoNOS) than controls (12.76 vs. 9.6, *p* ≤ 0.01). Patients on trazodone had a longer time between diagnosis and death than control patients (2.45 vs. 2.29 years), but this did not reach statistical significance.

Trazodone treatment in dementia is uncommon. Our data, combined with that of Sommerlad et al., covers the records of a population of over 4.5 million people and together we identify only 563 such patients (406 in Sommerlad et al, 157 in our study). Both studies reveal trazodone is used at relatively low doses (mean 75 mg), less than half the dose (200 mg) predicted to have a disease‐modifying effect from pre‐clinical research.^2^ Both studies found high levels of neuropsychiatric symptoms and treatment started relatively late in the disease course.

Observational studies cannot definitively answer the question as to whether an agent may be disease‐modifying. The preclinical evidence for the UPR as a therapeutic target remains compelling but at a stage in disease equivalent to earlier stages in humans.^2^, ^6^ The data from naturalistic cohorts inform only on patients with dementia with moderately advanced disease taking trazodone at low doses. They do not inform on any possible disease‐modifying effect of trazodone at higher doses, earlier in disease. This requires a randomized controlled trial. We believe it is premature to discard a promising therapeutic possibility based only on naturalistic observational studies where experimental medicine approaches are possible and which could inform any subsequent clinical trial to understand whether trazodone may be disease‐modifying in dementia.

## AUTHOR CONTRIBUTIONS

Benjamin R Underwood and Emad Sidhom had the concept for the study and collected and analyzed the data. Macky Stephen Padilla analyzed the data. Simon White provided statistical advice and Jonathan Lewis provided the custom codes to access the database. All authors contributed to writing and reviewing the manuscript.

## CONFLICT OF INTEREST

The authors declare no conflict of interests relevant to this article.

## Data Availability

Patient‐level data are not publicly available, under NHS Research Ethics terms. Source code and summary data are available on reasonable request.
